# Proteomic analysis of colon and rectal carcinoma using standard and customized databases

**DOI:** 10.1038/sdata.2015.22

**Published:** 2015-06-23

**Authors:** Robbert J.C. Slebos, Xia Wang, Xaojing Wang, Bing Zhang, David L. Tabb, Daniel C. Liebler

**Affiliations:** 1 Department of Biochemistry, Vanderbilt University School of Medicine, Nashville, TN 37232, USA; 2 Jim Ayers Institute for Precancer Detection and Diagnosis, Vanderbilt-Ingram Cancer Center, Nashville, TN 37232, USA; 3 Department of Mathematical Sciences, University of Cincinnati, Cincinnati, OH 45221, USA; 4 Department of Biomedical Informatics, Vanderbilt University School of Medicine, Nashville, TN 37232, USA

**Keywords:** Proteomics, Proteomic analysis, Rectal cancer, Colon cancer

## Abstract

Understanding proteomic differences underlying the different phenotypic classes of colon and rectal carcinoma is important and may eventually lead to a better assessment of clinical behavior of these cancers. We here present a comprehensive description of the proteomic data obtained from 90 colon and rectal carcinomas previously subjected to genomic analysis by The Cancer Genome Atlas (TCGA). Here, the primary instrument files and derived secondary data files are compiled and presented in forms that will allow further analyses of the biology of colon and rectal carcinoma. We also discuss new challenges in processing these large proteomic datasets for relevant proteins and protein variants.

## Background & Summary

Emerging collections of genomics data for human cancers present the formidable challenge of understanding how genomic abnormalities drive the biological and clinical characteristics of cancer. This task will be facilitated by proteomic analyses, which provides an intermediate layer of biological information that is more directly connected to phenotype. The National Cancer Institute (NCI) Clinical Proteomic Tumor Assessment Consortium (CPTAC) network employs proteomic technology platforms to analyze colorectal, breast and ovarian tumors previously analyzed by the NCI The Cancer Genome Atlas (TCGA) network, with the objective of integrating proteomic and genomic data for the same tissue specimens^1^. Here we describe datasets and data analysis methods for the proteomic analysis of colon and rectal tumors from the TCGA, which were combined with TCGA genomics data to produce the first integrated proteogenomic analysis of a human cancer^2^.

We analyzed 95 samples representing 90 TCGA colon and rectal (CRC) tumors, which comprise a subset of the 224 sample collection subjected to multiplatform genomic analyses by the TCGA^3^. Proteomic analyses by multidimensional liquid chromatography-tandem mass spectrometry (LC-MS/MS) provide peptide and protein identifications from MS/MS data and estimates of relative abundance, from which expression differences can be inferred^4^. Our data analysis employed a workflow that is well-established for global proteomics^4^, but is typically used for much smaller datasets. The large size of our proteomic dataset created unanticipated challenges and required additional evaluation of alternative analyses strategies. The final protein assembly summarizes a total of more than 13 million successfully assigned MS/MS spectra representing more than 7,000 non-redundant human protein identifications. We used this dataset to create a proteomics-based classification of colon and rectal carcinoma and demonstrate its use in relation to genomics-based classifications^2^. Chromosomal mapping of protein expression profiles revealed both *cis*- and *trans*-effects of chromosomal loci commonly involved in amplification and deletion events. Protein expression changes may be used to create a better understanding of the driving forces behind such chromosomal changes. In addition, we employed a novel strategy that integrates individual sequence variation as determined by RNA-seq with proteomic identification to improve protein identification and discover single amino acid variation (SAAVs) in the proteome. The current paper describes this dataset in detail and makes available additional files that will facilitate further analyses based on our proteomics data. These additional files include search results from the three different protein-search engines and customized databases containing all sequence variations for each of the 90 TCGA tumors. We also discuss the different options at each of the data processing steps and the consequences for the eventual protein inventory.

## Methods

### Summary

Description of data acquisition and processing were expanded from our previous work^2^. After receipt of the tumor blocks, protein was extracted from the samples, digested with trypsin, and the resulting peptides were fractionated by off-line basic-reverse phase high-performance liquid chromatography. Each tumor sample yielded 15 concatenated peptide fractions, which then were analyzed by reverse phase liquid chromatography-tandem mass spectrometry. Analysis of the 95 carcinoma and 60 normal epithelial samples took 9 months on a single MS instrument. To monitor system stability and performance, we also analyzed benchmark quality control (QC) samples from one basal and one luminal human breast tumor xenograft. These were analyzed in alternating order after every 5 sets of colorectal carcinoma and normal colon epithelium samples. This produced proteomics QC datasets for both the 16 basal and luminal xenografts (Data Citation 1). The QC dataset not only enabled assessment of platform performance and consistency throughout the project, but also provided a representative large proteomics dataset with which to evaluate data normalization. Raw MS/MS data were processed for peptide identification by database and spectral library searching using three different search engines (Myrimatch, Pepitome and MS-GF+)^5–7^. Identified peptides were assembled as proteins and mapped to gene identifiers for proteogenomic comparisons using IDPicker 3.0 (ref. 8). Quantitative comparisons were based on spectral count data. Details for each of the preparation steps are given below.

### TCGA tumor samples

All samples for the current study were obtained through the TCGA Biospecimen Core Resource (BCR) as described previously^3^. Of the 276 samples described in the TCGA study, 95 specimens from 90 patients were available for the current study (5 patients’s tumors were provided as duplicate pieces). No other selection criteria other than availability were applied for this study. Specimens for proteomic study were sectioned sequentially from the tumor blocks used for genomic studies, hence the ‘bottom’ slides from the genomic studies are representative of the material used for proteomics. The slides are available from the TCGA data portal (http://tcga-data.nci.nih.gov/tcga/tcgaHome2.jsp). All samples were frozen within 1 h of collection and histopathological examination confirmed that they contained at least 60% tumor nuclei as described earlier^3^. Prior CPTAC studies have shown that global protein levels, but not phosphorylation states, are stable within this 1 h time period^9^. Samples were shipped from the TCGA BCR on dry ice and kept frozen at −80 °C until processing. All TCGA colorectal tissue samples were washed prior to digestion to eliminate any residual optimal cutting temperature compound (OCT). The tissue was placed in a 1.5 ml micro-tube was washed with 1 ml 70% EtOH/30% H_2_O for 30 s with vortexing. The supernatant was then discarded and the tissue was washed with 1 ml of 100% H_2_O 30 s with vortexing and again the supernatant was discarded. One milliliter of 70% EtOH/30% H_2_O was added to the tissue sample and incubated for 5 min at room temperature following by centrifugation at 20,000×g for 2 min at 20 °C. The supernatant was removed, and this wash step was repeated. Next, 1 ml of 85% EtOH/15% H_2_O was added to the tissue and incubated for 5 min at room temperature following by centrifugation at 20,000×g for 2 min at 20 °C. The supernatant was removed, and the wash was repeated. For the final wash, the tissue was washed in 1 ml 100% EtOH, incubated 5 min at room temperature, and centrifuged at 20,000×g for 2 min at 20 °C. The supernatant was removed, and the final wash was repeated.

### Normal colon epithelium

Normal colon epithelium biopsies were obtained through the Cooperative Human Tissue Network (CHTN) Western Division at Vanderbilt University. Samples were collected from screening colonoscopies performed between July 2006 and October 2010 under Vanderbilt University IRB approval #061096. During colonoscopy, multiple pinch biopsies were obtained from both ascending and descending colon and immediately frozen in liquid nitrogen. Biopsies obtained from 30 subjects with completely normal findings during colonoscopy were included in the study. A total of 60 specimens, one from ascending and one from descending colon, were used for proteomic analysis.

### Protein extraction and digestion of tissue specimens

Following OCT removal, tissue specimens were placed in 1.5 ml micro-centrifuge tubes and re-suspended 100 μl of trifluoroethanol (TFE) and 100 μl of 100 mM ammonium bicarbonate, pH 8. If additional buffer was required, equal volumes of TFE and 100 mM ammonium bicarbonate pH 8.0 were added accordingly. In addition, powderized xenograft tumor tissue representing the Comparison/Reference (CompRef) samples for Luminal (WHIM16) and Basal (WHIM2) breast cancer subtypes were analyzed in alternating order after every 5 TCGA colorectal tissue samples. Samples were sonicated using a Fisher Scientific Sonic Dismembrator Model 100 at a setting of 20 watts for 20 s followed by 30 s incubation on ice. This sonication step was repeated twice, and samples were placed on ice between sonications. The resulting homogenate was heated with shaking at 1,000 rpm for 1 h at 60 °C followed by a second series of sonication steps, as described above. A protein measurement then was obtained for each sample using the BCA Protein Assay (ThermoFisher Pierce, Rockford, IL) using the manufacturer’s protocol.

An aliquot equivalent to 200 μg was removed and reduced with tris(2-carboxyethyl)phosphine (TCEP, 20 mM) and dithiothreitol (DTT, 50 mM) at 60 °C for 30 min, followed by alkylation with iodoacetamide (IAM, 100 mM) in the dark at room temperature for 20 min. The lysate was diluted with the appropriate volume of 50 mM ammonium bicarbonate, pH 8.0, to reduce the TFE concentration to 10%. Trypsin was added at a ratio of 1:50 (w:w) and digested overnight at 37 °C. The digested mixture was frozen at −80 °C and lyophilized to dryness. The lyophilized samples were re-suspended in 350 μl of HPLC-grade water and vortexed vigorously for one minute and desalted using an Oasis HLB 96-well μElution plate (30 μm, 5 mg, Waters Corp., Milford, MA), which was pre-washed with 500 μl of acetonitrile and equilibrated with 750 μl of HPLC-grade water. The flow-through was discarded, and the plates were washed with 500 μl of HPLC-grade water. The peptides were eluted with 80% acetonitrile, and the eluates were evaporated to dryness in vacuo. Samples were stored in the freezer until further analysis.

### Peptide fractionation by basic reverse phase liquid chromatography (bRPLC)

Samples were reconstituted in 300 μl of Solvent A (1.0 M triethylamine bicarbonate (TEAB), pH 7.5). The reconstituted sample was then diluted with an additional 100 μl of Solvent A and the entire 400 μl of solution was injected into the bRPLC column. Tryptic peptides were fractionated using bRPLC separation with an XBridge BEH C18, 250 mm×4.6 mm analytical column (130A, 5 μm particle size) equipped with a XBridge BEH C18 Sentry guard cartridge at a flow rate of 0.5 ml/min. The solvents were 10 mM TEAB pH 7.5 in water as mobile phase A and 100% acetonitrile as mobile phase B. Sample fractionation was accomplished using the following multistep linear gradient: from 0 to 5% B in 10 min, from 5 to 35% B in 60 min, from 35–70% in 15 min and held at 70% B for an additional 10 min before returning to initial conditions. A total of 60 fractions were collected over the 105 min gradient and concatenated into 15 fractions by combining fractions 1, 16, 31, 46; 2, 17, 32, 47; and so on up to fractions 15, 30, 45, and 60. The samples were evaporated to dryness in a Speed-Vac sample concentrator and stored at −80C until LC-MS/MS analysis.

### LC-MS/MS analysis

Resulting peptide fractions were resuspended in 50 μl of 2% acetonitrile/0.1% formic acid and analyzed using a Thermo LTQ Orbitrap Velos ion trap mass spectrometer equipped with an Eksigent NanoLC 2D pump and AS-1 autosampler. A 2 μl injection volume of the peptide solution was separated on a packed capillary tip (Polymicro Technologies, 100 μm×11 cm) containing Jupiter C18 resin (5 μm, 300 Å, Phenomenex) using an in-line solid-phase extraction column (100 μm×6 cm) packed with the same C18 resin using a frit generated with liquid silicate Kasil^10^. Mobile phase A consisted of 0.1% formic acid and Mobile phase B consisted of 0.1% formic acid in acetonitrile. A 95 min gradient was preceded by a 15 min washing period (100% A) at a flow-rate of 1.5 μl min^−1^ to remove residual salt. Following the wash, the mobile phase was programmed to 25% B by 50 min, followed by an increase to 90% B by 65 min and held for 9 min before returning to the initial conditions. A full MS scan was collected for peptides from 400–2000 m/z on the Orbitrap at a resolution of 60,000 followed by eight data-dependent MS/MS scans from lowest to highest signal intensity on the LTQ. Centroided MS/MS scans were acquired using an isolation width of 3 m/z, an activation time of 30 ms, an activation q of 0.250 and 35% normalized collision energy. One microscan with a max ion time of 100 and 1000 ms was used for each MS/MS and full MS scan, respectively. MS/MS spectra were collected using a dynamic exclusion of 60 s with a repeat of 1 and repeat duration of 1.

All TCGA and normal colon epithelial samples were analyzed on the same Thermo LTQ Orbitrap Velos instrument, with sample analysis beginning on July 26, 2012 and concluding on June 27, 2013. Each sample comprised the 15 bRPLC fractions prepared as described above. Benchmark quality control (QC) samples from one basal and one luminal human breast tumor xenograft (Comparison/Reference, ‘CompRef’ samples) were analyzed in alternating order after every five CRC samples. Specifically, five TCGA samples were run on the instrument, followed by a luminal CompRef sample, and then another five TCGA samples followed by a basal CompRef sample for a total of 32 CompRef samples. Normal epithelial samples were analyzed using the same schema whereby each 5 normal epithelial samples were followed by a CompRef sample. Bovine serum albumin (BSA) tryptic digest standards were analyzed before and after every ten TCGA samples and were used to monitor instrument sensitivity, BSA standard sequence coverage and chromatographic performance to determine acceptance or rejection of the acquired data.

### LC-MS/MS peptide identification

Peptide identification employed the RefSeq Human protein sequence database, release version 54, and both database and peptide library search strategies. Two bovine trypsin sequences and one porcine trypsin sequence were appended to these 34,586 sequences. The June 14, 2011 NIST human spectral library for ion traps (617,000 spectra, counting paired decoys) was indexed against this sequence database. Thermo RAW files were converted to mzML peaklists by the ScanSifter algorithm^11^ or by ProteoWizard msConvert^12^. The ScanSifter files were employed by Pepitome 1.0.42 (ref. 6) for spectral library search and MyriMatch 2.1.87 (ref. 5) for database search, while the msConvert files were used by MS-GF+ v9176 (ref. 7). Pepitome and MyriMatch employed precursor tolerances of 10 ppm, while MS-GF+ used a 20 ppm window; all three algorithms allowed fragments to vary by up to 0.5 m/z, and both database search engines considered semi-tryptic peptides equally with fully-tryptic peptides, allowed for isotopic error in precursor ion selection, conducted on-the-fly peptide sequence reversal, and applied static +57 modifications to cysteines and dynamic +16 oxidations to methionines. MS-GF+ considered acetylation for protein N-termini, while MyriMatch added pyroglutamine modifications to the N-termini of peptides starting with Gln residues. Pepitome considered any modification variants and trypsin specificities that were included in the spectral library.

### Protein assembly

Spectral identification files from each of the search engines (pepXML for Myrimatch and Pepitome, mzIdent for MS-GF+) were converted to IDPicker 3 SQLite databases (idpDB) using IDP3 (ref. 8). The resulting 4,275 idpDB files (95 samples×15 fractions×3 search engines) were used for a final protein assembly using IDP3. For initial protein assembly, peptide identification stringency was set at a maximum of 1% reversed peptide matches, i.e., 2% peptide-to-spectrum matches (PSM) FDR and a minimum of 2 distinct peptides to identify a given protein within the full data set. To optimize the number of proteins identified, we applied a very stringent filter at 0.1% PSM FDR and a minimum of 2 distinct peptides identified for each protein. This filter resulted in the identification of a total of 94,442 distinct peptides among the 95 samples representing 7,526 protein groups with a protein-level FDR of 2.64%. To rescue high quality PSMs that were excluded by the stringent PSM FDR threshold, we relaxed the PSM FDR threshold to 1% for the confidently identified proteins. This step increased the number of distinct peptides identified to 124,823, corresponding to a total number of 6,299,756 spectra; the rescued PSMs were of high quality, with a median PSM FDR of less than 0.2%, confirming that the integrity of the data set was maintained. To facilitate the integration between genomic and proteomic data, we further assembled the peptides at the gene level. This assembly resulted in 7,211 gene groups with a gene-level FDR of 2.7%. Genes identified from each sample ranged from 3,372 to 5,456, with a median gene count of 4,656 for the 95 samples. 1,530 genes (21%) were found in all 95 samples, 4,628 genes (64%) in more than half of the samples, and only 10 genes (0.1%) in just a single sample. In addition, we created a combined protein assembly using all TCGA colorectal carcinoma and the 60 normal colon epithelium samples. This assembly identified 7,548 protein groups with a protein FDR of 2.7%. All IDP3 protein assembly files are available through ProteomeXchange (Data Citation 13) or from the CPTAC data portal (cptac-data-portal.georgetown.edu/cptacPublic).

### Customized database construction

We developed an R package *customProDB* ^13^ to annotate variations predicted from RNA-Seq, including mapping to dbSNP135 and COSMIC64 databases. For each sample, *customProDB* generates a protein FASTA database by appending proteins with nonsynonymous protein coding single nucleotide variants (SNVs) and aberrant proteins to the end of the standard RefSeq Human protein sequence database. Due to the low coverage of this RNA-Seq data set, we did not remove the low abundance transcripts from the standard RefSeq database. Peptide identification was performed for each sample separately using the corresponding customized FASTA database with MyriMatch 2.1.87. IDPicker 3 was produced a conjoint protein assembly across all the customized database searches, with filtering set at 1% PSM FDR and a minimum of 5 spectra identified per protein. The full data set consisted of 8,352 protein groups with 1.8% protein FDR. Identified single amino acid variations (SAAVs) were further annotated from the previous report of the TCGA study^3^ (i.e., TCGA-somatic variants), existence in the COSMIC64 database (i.e., COSMIC-supported variants), and existence in the dbSNP135 database (i.e., dbSNP-supported variants). To identify TCGA-somatic variants, we downloaded the MAF (Mutation Annotation Format) files from the FIREHOSE website (Data Citation 2 and Data Citation 3). Since the coordinates in MAF files were based on hg18, liftOver (http://hgdownload.cse.ucsc.edu/admin/exe/) from UCSC was used to convert genome coordinates to hg19. All identified variant peptides as well as SAAVs and their annotations can be found in the original publication^2^.

### Variant peptide identification

To identify variant peptides, we performed database searches using the above-constructed sample-specific customized databases as previously described^14^. Identifying SNVs and INDELs from RNA-Seq data BAM files for 86 of the 90 patients were downloaded from CGHub in February 2013. Although 87 samples were analyzed by RNA-Seq, we were unable to obtain the BAM file for one of the 87 samples. Tophat (v2.0.7) was used to re-align reads to human reference genome (hg19) in a spliced mode using default parameters, allowing a maximum of 10 hits per read. The resulting BAM files were indexed using samtools (0.1.18, http://samtools.sourceforge.net/). We used an in-house script to summarize the reads mapping information and calculate the exon coverage. Seventy-six percent of exons were covered by RNA-Seq reads, and 64% had an average coverage greater than 1. Putative single nucleotide variants (SNVs) and short INDELs were called one library at a time using mpileup and varFilter from samtools. Putative SNVs were further filtered based on the following criteria: (i) SNP quality≥20; (ii) mapping quality≥20; and (iii) read depth≥3 and then put into a VCF file. For short INDEL candidates, gapped reads≥3 were required.

### Protein quantification

We used spectral count, or the total number of MS/MS spectra acquired for peptides from a given protein in a given LC/LC−MS/MS analysis, as the basis for protein quantification. Spectral count is linearly correlated with the protein abundance over a large dynamic range^15^. This simple but practical quantification method has found broad application in detecting differential or correlated protein expression^16–19^, and multiple groups have concluded that spectral counting achieves similar accuracy to more complex methods such as the intensity-based techniques^19–21^. Previously, we have confirmed proteomic changes detected from spectral count data by targeted proteomics with multiple reaction monitoring (MRM) in different data sets^22,23^.

For the CompRef QC sample data set, spectral count data were summarized at the protein group level, where a protein group is defined as the set of proteins that are indiscernible on the basis of the observed peptides. For each group, a random protein was selected to represent the group. The spectral count table has data for 8,029 mouse/human protein groups from 32 samples, with 16 basal samples and 16 luminal samples. For the TCGA tumor data set, to facilitate the integration between genomic and proteomic data, spectral count data were summarized at the gene group level, where a gene group is defined as the set of genes that are indiscernible on the basis of the observed peptides. To ensure reproducibility, the longest protein was selected for each gene in the calculation of protein length. For each gene group, the gene with the shortest protein length was selected to represent the group following the Occam’s razor principle. The final spectral count Table has data for 7,211 genes and 95 samples (5 tumors have duplicated samples, Supplementary Table S1). For analyses that require only one sample from the duplicates, the sample with a larger total spectral count was selected.

### Quality control using QuaMeter

The raw data for each of the 95 bRPLC experiments were subjected to quality control assessment in the QuaMeter ‘IDFree’ mode^24^, producing forty-four metrics for each file along with its timestamp. Metrics included peak height and width distributions, MS scan rates, precursor charge states, and total ion current distributions, and identifications were not a factor in any of the metrics. The table of metrics included 1,425 rows, one for each RAW file (Supplementary Table S2).

Scripts were created in the R statistical environment for multivariate analysis of these metrics. The software performed a robust Principal Components Analysis (PCA) on the values, excluding the following fields due to insufficient variation: XIC-FWHM.Q1, XIC.FWHM.Q3, RT.Duration, and all precursor charge state fields. In PC space, the normalized Euclidean distance between pairs of RAW files was computed for the fifteen files produced for each bRPLC. When a LC-MS/MS experiment yields a large median standard distance from the other files, that distance suggests the experiment is an outlier in QC. When all the files of a bRPLC experiment have high median distances, they are indicative of high variability in instrument performance for that sample.

### Code availability

Exact copies of all open source software, customized scripts and data processing procedures are provided through the ProteomeXchange Consortium via the PRIDE partner repository as 'other' files (Data Citation 13).

## Data Records

### Primary MS instrument and database search files

Primary files for this study are in the Thermo ‘RAW’ file format accessible through the ProteomeXchange repository (Data Citation 1 and Data Citation 4, Data Citation 5, Data Citation 6, Data Citation 7, Data Citation 8, Data Citation 9, Data Citation 10, Data Citation 11, Data Citation 12, Data Citation 13 and Data Citation 14) or from the CPTAC data portal (cptac-data-portal.georgetown.edu/cptacPublic). Included are data files from the 95 TCGA carcinoma samples, the 60 normal epithelial biopsy samples and the CompRef control samples. A complete list of files is provided in Supplementary Table S3. Protein identification from these files was accomplished through database searching using Myrimatch and MS-GF+, and spectral library searching using Pepitome (fully human samples only). Resulting search files are in the pepXML file format for Myrimatch and Pepitome searches and in the mzID file format for MS-GF+ searches. Output files from all searches using the standard human RefSeq database are provided in a single compressed file. Likewise, all search results from normal epithelium and search results using each of the customized databases are summarized in single compressed files. These databases are available from the CPTAC data portal. In addition to the IDPicker 3 (IDP3) protein assembly file combining search results from the original publication^2^, we here present IDP3 files from each of the individual search engines and the IDP3 file containing only the normal epithelium control samples. IDP3 is an open-source protein assembly and filtering tool that queries search results summarized in a SQLite database format^8^. The protein databases against which all searches were performed are provided separately: 1) the standard human database file (RefSeq version 54), 2) the accompanying standard spectral library file, 3) a single compressed file containing each of the 86 customized databases that include the standard search information plus all variant sequences identified in TCGA RNA-seq data from the CPTAC data portal, and 4) the combined human/mouse RefSeq database. The processing and result files have been deposited to the ProteomeXchange Consortium via the PRIDE partner repository (Data Citation 1, Data Citation 13 and Data Citation 14).

### Data processing strategies

**Figure 1:**
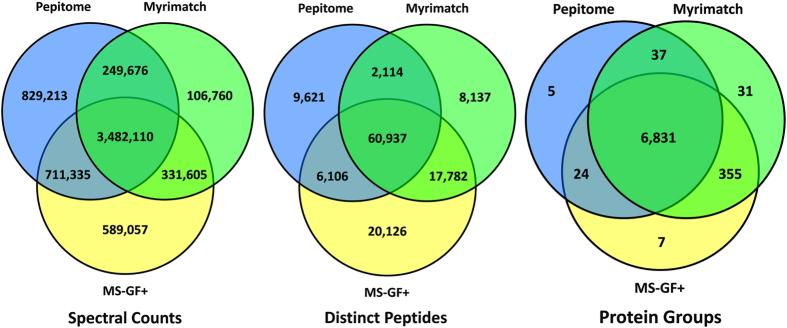
Comparison of identified spectra, peptides and proteins by the three search engines Myrimatch, Pepitome and MS-GF+. Over 93% of the proteins in the dataset are identified by all 3 search engines, while the spectra and peptide inventories benefit more from each of the individual contributions of the different search engines. The spectral library search engine Pepitome increases the overall spectral count totals by 13% through the identification of previously observed spectra that were not identified by the other search engines. The contribution of Pepitome is less in the area of unique peptides and proteins. MS-GF+ contributes a large fraction of all spectral counts (9.4%) and distinct peptides (16.1%).

One of the goals of bioinformatics analysis of shotgun proteomic dataset is to maximize the number of successfully identified MS/MS spectra without compromising the quality of the data. Almost all spectrum identification strategies are based on comparing obtained spectra with predicted spectra from known amino acid peptide sequences (‘database searching’) or with previously observed and identified spectra (‘spectral library searching’) (for review see^25^). Over the years, several algorithms have been developed that accomplish this goal with varying levels of sophistication, and these have been incorporated into commercially available or open-source software tools for peptide identification. For instance, most algorithms calculate from known amino acid masses where one would expect peptide fragments to appear on a MS/MS spectrum, but not all would take signal intensity into account when matching to the observed spectrum. Newer algorithms also take complementarity of the fragment ions into account (e.g., MS-GF+), looking for the remaining part of the precursor mass when a given peptide fragment is observed. A commonly accepted strategy to avoid false-positive matches is to include distractor (decoy) sequences in the database and to set limits on the proportion of such decoy matches in the final list of identified peptides^26^. These distractor sequences can be generated by adding reversed amino acid sequences in the database (reverse-peptides), an approach that has the advantage of preserving the distribution of precursor masses in the (reversed) search space. The various strategies in search algorithms cause differences in their identification profiles and it is helpful to combine search results from multiple search engines to come to an optimal annotation of a given set of MS/MS spectra^27^. For this study, we chose three open-source search tools (Myrimatch, MS-GF+ and Pepitome^7,28^) and combined their results into a single protein assembly using IDP3. Figure 1 shows the distribution of spectra and protein group identifications between the three different search engines. Note that even though the number of identified proteins is not greatly increased by combining the data, each of the search engines contributes a significant number of engine-specific spectral matches. The spectral library search engine Pepitome matches spectra to previously identified spectra from the library and is better at finding such known spectra than the other two search engines. This leads to higher numbers of spectral counts, but not necessarily of distinct peptides or protein groups. On the other hand, MS-GF+ identifies a distinct group of additional peptide-spectrum matches, which leads to a large number of added counts from distinct peptides uniquely discovered by this search engine. These results indicate that it is important to combine results from different approaches to increase the variety and number of identifications and thus the universe of proteins that are quantifiable by spectral counting.

IDP3 uses an inclusive combination algorithm whereby all PSMs that pass filtering criteria for an individual search engine are included in the assembly, using the highest scoring PSM from the search engines. IDP3 uses protein parsimony filtering to assemble identified peptide lists into a list of non-redundant proteins (‘protein groups’) as described previously^29^. To allow easy comparison to genomic data, IDP3 layers genome data on top of the RefSeq protein identifications, so that the main output becomes a non-redundant list of RefSeq gene IDs (‘gene groups’) for which peptide evidence was obtained. The 95 TCGA colon cancer specimens yielded a dataset of 7,526 protein groups representing 7,211 gene groups.

### Cut off points for filtering, stringencies PSM/peptide/protein FDR

The goal of our bioinformatics analysis of the TCGA colorectal cancer dataset was to maximize the number and diversity of identified peptides without sacrificing confidence in the obtained spectral matches. A common strategy to determine false-positive rates in shotgun proteomics datasets is to include decoy sequences in the database. Decoy sequence identifications enable modeling of the score distribution for false matches in a given dataset. The maximum allowable fraction of decoy peptide-spectrum matches in shotgun proteomic datasets is commonly set at 1 or 2%, although these are arbitrarily chosen cut-off values. In addition, the requirement of having at least 2 distinct peptide sequences for the identification of a single protein can also limit the number of falsely identified proteins. However, applying these two criteria may not be sufficient to limit the number of falsely identified proteins in a dataset, as many studies do not report protein FDR values in separate calculations. This is especially true with larger protein assemblies where the vast majority of unique peptide sequences have been identified already and any additional peptide identifications are therefore more likely to be false. One strategy we employed in moderately large datasets is to increase the minimum number of matched spectra required for protein acceptance into the assembly^22,23^. This strategy works well and protects against inclusion of proteins that have little spectral evidence, as well as against false-positive peptide identifications. The drawbacks of using a minimum number of spectral counts per protein are that low-abundance proteins may be missed and that the number of identified proteins is greatly reduced. In very large datasets, such as the one reported here, proteins with high expression in just a few samples also may be missed. With the ever increasing size of shotgun experiments (our TCGA colorectal carcinoma assembly was based on 4,275 search result files), limiting PSM FDR and spectral counts is not sufficient to limit false-discovery rates to acceptable levels.

**Figure 2:**
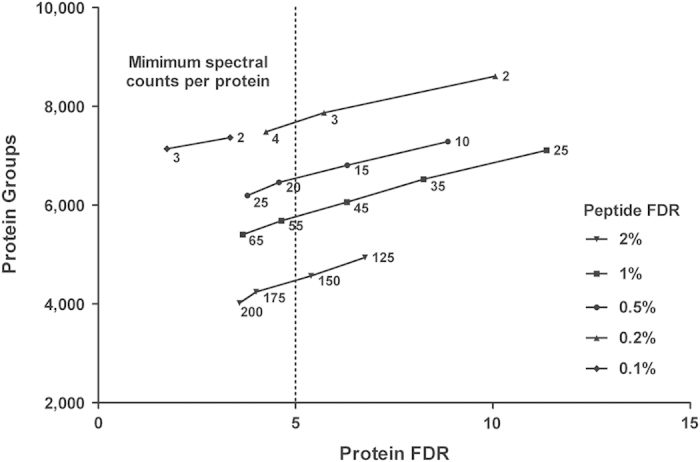
Impact of peptide-to-spectrum match (PSM) false discovery rate (FDR) threshold at different levels of overall protein FDR on the total protein inventories. Surprisingly, increased PSM FDR stringency (lower values) increased the number of identifiable proteins. Protein FDR was maintained below 5% by requiring a minimum number of spectral counts for each protein across the dataset. The spectral count minimum requires to maintain a protein FDR below 5% increased with the applied PSM FDR.

To deal with the false discovery challenge in our large dataset, we developed a new strategy based on the counter-intuitive finding that increasing the stringency of PSMs actually increased the number of proteins identified. This is illustrated in Fig. 2, which presents the number of protein groups identified at different PSM and protein false-discovery rates at different minimum numbers of spectral counts. The lower curve shows the values obtained at the commonly accepted PSM value of 2%, where a minimum number of spectral counts per protein needs to be set at 175 to achieve a protein FDR of less than 5%. Because of the loss of a large number of proteins with lower numbers of spectral counts, this setting also yields the lowest number of protein groups identified (~4,000). When PSM FDR rate is set more stringently, lower-cut off values for the minimum number of spectral counts per protein can be selected without sacrificing protein FDR. This leads to higher numbers of identified protein groups at an acceptable protein FDR, albeit with lower spectral counts per protein than using the more lenient PSM FDR settings.

Based on these considerations, we adopted a 2-step procedure, whereby we initially set a very stringent PSM FDR of 0.1%, which maximized the number of identified protein groups at an acceptable protein FDR. This defined the inventory of proteins that could be confidently identified in this dataset. The second step was then to increase the PSM FDR 10-fold to 1% for only the protein groups already identified using the more stringent setting. Thus, additional PSMs could be rescued without adversely affecting the protein FDR, which remained locked at the level from the first step of the procedure. Examination of the rescued peptides revealed that the majority had PSM FDR well below acceptable levels (median 0.2%), thus demonstrating that these additional peptide matches did not compromise the accuracy of the protein identifications^2^. Our analysis of this dataset illustrates challenges associated with very large proteomic studies. Although our approach adapts established target-decoy methodology, new approaches to this challenge merit further study.

### Detection of novel single amino acid variants (SAAVs) in the dataset

We previously designed and implemented a new strategy for the detection of protein variant sequences by using genomic data for peptide identification^14,22^. This new approach was used to leverage mRNA sequencing data for the discovery of 796 SAAVs in the TCGA colorectal carcinoma dataset. About 70% (561 of 796) of these had been previously described as single nucleotide polymorphisms (SNPs) occurring as natural variation in the human population. SNPs leading to amino acid changes in proteins occur with varying frequencies and are generally not associated with disease phenotypes^30^. Of the remaining 235 SAAVs, 73 were previously described in either the COSMIC database or tagged as somatic mutation in the genomic analysis of the TCGA dataset^2^. Thus, the remaining 162 SAAVs detected in our analysis could not be explained by existing knowledge and were determined to be ‘new’ (Supplementary Table S4). Given the low false-discovery rate in our proteomic dataset and the fact that independent, RNAseq-based, evidence existed, the likelihood that these new SAAVs were real findings was high. To determine the reliability of the identifications we manually examined the top SAAVs with at least two spectra identified (*N*=70) from the dataset using BLAST searches and matching to the most recent National Center for Biotechnology Information (NCBI) database of SNPs.

The results from this follow up analysis demonstrate that many of the most abundant of these SAAVs are correct assignments, which appear to be truly novel (summarized in Supplementary Table S4). The analyses also revealed apparently trivial explanations for some incorrect assignments, which nevertheless are instructive. For example, peptide ‘INLVQR’, representing the F76I variant of DWR67 with the highest number of spectral counts (375) is an isobar, indistinguishable by mass spectrometry from the peptide ‘LNLVQR’ from LDHA, an abundant metabolic protein. Three other peptides had a similar L to I variation which, given the RNA-seq data, were likely to be true but could not be confirmed by MS because of the lack of a mass difference. There were 14 peptides that matched to SNPs not present in the original databases and 6 peptides that matched to published immunoglobulin and major histocompatability complex sequence variants. The remaining 46 of the originally selected 70 peptides appear to be truly novel. Two variant sequences each were found for 2 proteins (MYH9 and PKM), resulting in a total of 44 proteins with novel variant sequences. Future research will be needed to examine the biological relevance of each of the observed variant sequences. Furthermore, these results underscore the need to perform additional manual analyses on variant peptides identified through automated procedures as recently reviewed by Nesvizhski^31^. These include manual inspection for trivial explanations, comparisons to sequence databases and comparison with chromatography and spectra obtained from synthetic variant sequence peptides.

### Germline versus somatic single amino acid variations

One observation we originally made on the basis of SAAV detection in 10 colorectal carcinoma cell lines was that germline variants were about twice as likely to be detectable at the protein level as somatic variants^22^. The larger set of TCGA colorectal carcinomas provided a second confirmation of this original observation, again, the proportion of germline variant sequences observed in the mass spectrometry data was about twice as high as the proportion of somatic variant sequences that would have been expected based on their abundance in the full dataset^2^. These numbers are potentially influenced by the limitations of detection by mass spectrometry, although those limitations apply to both types of protein variants. Somatic variants may also be present at lower levels than germline variants because they are expected to only occur in the tumor cells and not in adjacent normal cells that also contribute to the overall proteomic inventory from the TCGA tumors. It is also possible that somatic variants are only present in a sub-set of the tumor cells due to tumor heterogeneity. However, both lines of reasoning do not apply to our earlier observation in the more homogeneous colorectal carcinoma cell lines. A direct comparison of germline- and somatically-derived variant proteins will be needed to confirm the validity of our original observations.

## Technical Validation

### Summary

We used three quality control strategies throughout the analysis of TCGA colorectal carcinoma samples. The first utilized a standard BSA tryptic digest after every colon carcinoma sample of 15 fractions, followed by manual inspection to assess peptide peak intensities and chromatographic features. The second was achieved by analyzing a CompRef^2^ sample after each set of 5 colorectal carcinoma samples and the third was by utilizing the identification-independent quality metric software package QuaMeter^24^.

### Day-to-day quality control

The BSA runs were evaluated by mass calibration check and using intensity and coverage of the BSA PSMs identified by database search. Mass axis shift was determined by comparing the experimental mass to the known monoisotopic mass of five different peptides found in the BSA standard. These peptides are: SLHTLFGDELCK (z=3), LVNELTEFAK (z=2), HLVDEPQNLIK (z=2), YICDNQDTISSK (z=2), KVPQVSTPTLVEVSR (z=2 and z=3). If the mass axis shift exceeded 10 ppm, the instrument was recalibrated prior to any sample runs. In addition, the BSA base peak chromatogram was viewed to determine that peak shape and retention times are appropriate. Specifically, a total intensity of 10^7^ derived from the BSA chromatogram and BSA coverage of at least 65% was required for acceptance of a set of colon carcinoma runs. Any failure to achieve these quality control measures prompted instrument maintenance before data acquisition was allowed to continue.

### Use of standardized CompRef sample

CompRef samples obtained from WHIM2 (basal type) and WHIM16 (luminal type) breast carcinoma xenografts^32^ were alternated and analyzed after every 5 colorectal carcinoma and normal epithelium samples throughout the full analysis period of the data set (from July 2012 until August 2013). Each of these runs was searched using MyriMatch against a combined human/mouse RefSeq-based database and the results compared to the initial CompRef samples used to establish our shotgun proteomic platform (not published). QuaMeter was used as a tool to evaluate instrument performance for each sample run. The quality control goals for each of these new runs were: 1) more than 5,000 identified spectra per run, 2) third quartile MS2 density of more than 200, and 3) more than 40% of identified spectra have a charge state of 2. These metrics guarantee that sufficient numbers of scans are detected, that they have sufficient signal to allow identification, and that stable precursor charge state ratios were achieved^24^.

**Figure 3:**
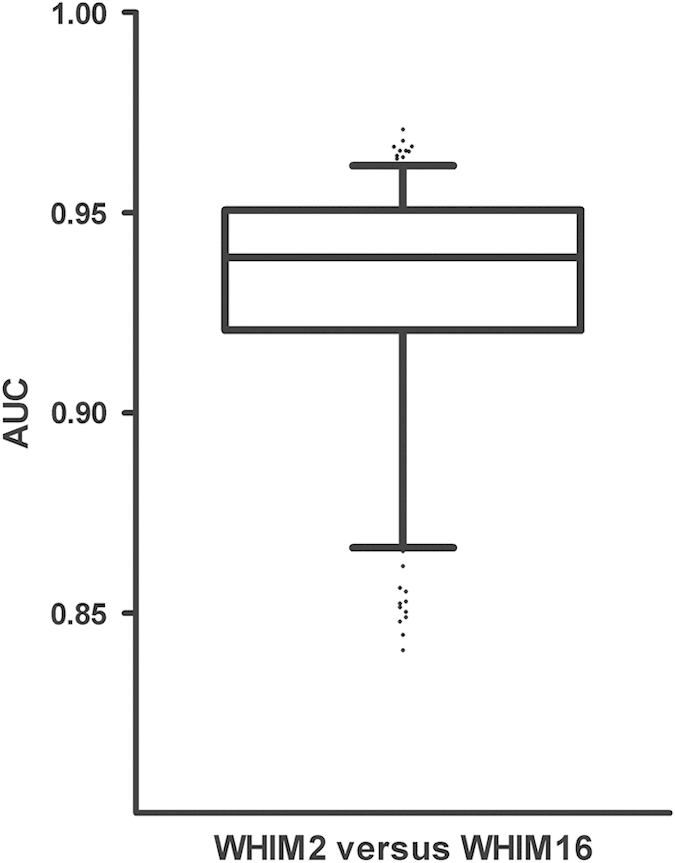
Performance of a 172 protein signature chosen to distinguish proteomic differences between single pairs of basal and luminal breast cancer xenografts. The protein signature distinguished the two breast tumor subtypes in almost all paired combinations of proteomic datasets generated for the two types of xenografts.

One of the main reasons to include the CompRef samples in this dataset was to compare the consistency of the observed biological differences between the basal and luminal subtypes. To test the consistency of such observations, we compiled a dataset containing all 32 CompRef samples that were analyzed with the TCGA carcinomas and normal biopsies. This dataset included a total of 8,029 identified protein groups with a protein FDR of 3.7% using a combined human/mouse RefSeq database (provided as an IDP3 database available through ProteomeXchange (Data Citation 13) or from the CPTAC data portal (cptac-data-portal.georgetown.edu/cptacPublic)). We compared the WHIM2 basal with the WHIM16 luminal samples using QuasiTel and selected 172 human proteins that distinguished the two xenograft types. Spectral counts for these proteins were at least 32-fold higher in one group over the other with an FDR- of less than 0.001. The full dataset for this analysis consisted of 5,633 proteins that had at least 45 counts across all 32 samples, according to the 1.4 spectral count average we earlier established to be necessary for appropriate quantification by spectral counting^2^. We then created comparisons for all 256 possible combinations of WHIM2 and WHIM16 (16×16 samples) between single pairs of the WHIM2 and WHIM16 samples and obtained spectral count ratios for all proteins. The predictive value of the set of 172 differential proteins (the ‘proteomic signature’) was tested against these combinations using Receiver Operating Characteristic (ROC) analysis using ‘area under the curve’ (AUC) as a measure of accuracy. The median AUC for this analysis was 0.931 (+s.d.=0.028), indicating that the most prominent differential proteins are captured when comparing single runs from each of the CompRef subtypes (Fig. 3). This suggests that biologically relevant phenotype differences remain observable even when individual MS analysis results are used.

### Use of instrument values for quality control

**Figure 4:**
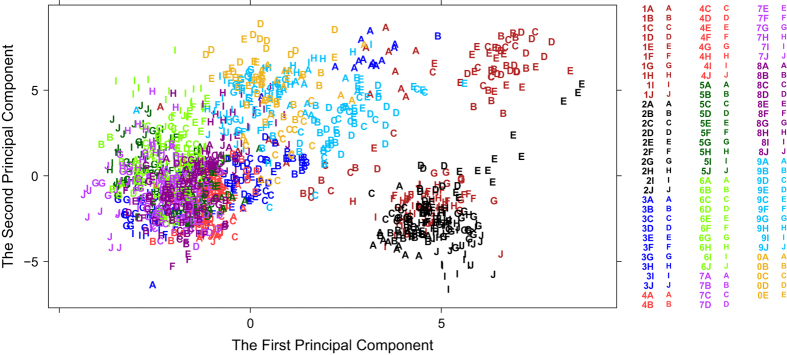
Principal component analysis of LC-MS/MS system performance metrics. Forty-four metrics from a total of 1,425 LC-MS/MS experiments were collapsed into two principal components, which accounted for 42.5% of the total variation.

The QuaMeter performance metrics have been computed for the full set of 1425 LC-MS/MS experiments for the 95 TCGA colon samples (see Methods). The software computed quality metrics for each file that were independent of identifications from Pepitome or any other search engine. These metrics enabled recognition of outliers among LC-MS/MS experiments for a particular sample and of batch effects from instrument drift over the ten-month interval during which these experiments were conducted. The 95 samples were run sequentially and are separated into 10 ‘batches’ for presentation purposes, with each sample represented by fifteen LC-MS/MS experiments. Each batch is represented by a different color in Fig. 4, with the ten samples each represented by a letter from A to J. QuaMeter reduced each LC-MS/MS experiment to a vector of 44 metrics, and multivariate analysis in the R statistical environment transformed the metrics by robust Principal Components Analysis (PCA). The letters in each batch generally occupy the same region of the principal component space. The first two principal components accounted for 42.5% of the variability (Fig. 4), suggesting diversity in the forces causing variation. The first principal component placed the most positive loading on extracted ion chromatogram height ratios (XIC.Height.Q2, XIC.Height.Q3) and the most negative loading on the number of peaks in MS/MS scans (MS2.Density.Q*). Component two, on the other hand, emphasized positive loadings on the retention time interval during which the second quartile of MS scans were acquired (RT.MS.Q2) and a negative loading on the third quartile of MS scan intensity (MS1.TIC.Q3) and of MS scan intensity change (MS1.TIC.Change.Q3). The lack of a clear-cut source of variation indicates that multiple, interacting components determine quality metrics stability, and thus do not point towards any particular part of the procedure that could be stabilized further to improve the quality metrics as determined by QuaMeter.

**Figure 5:**
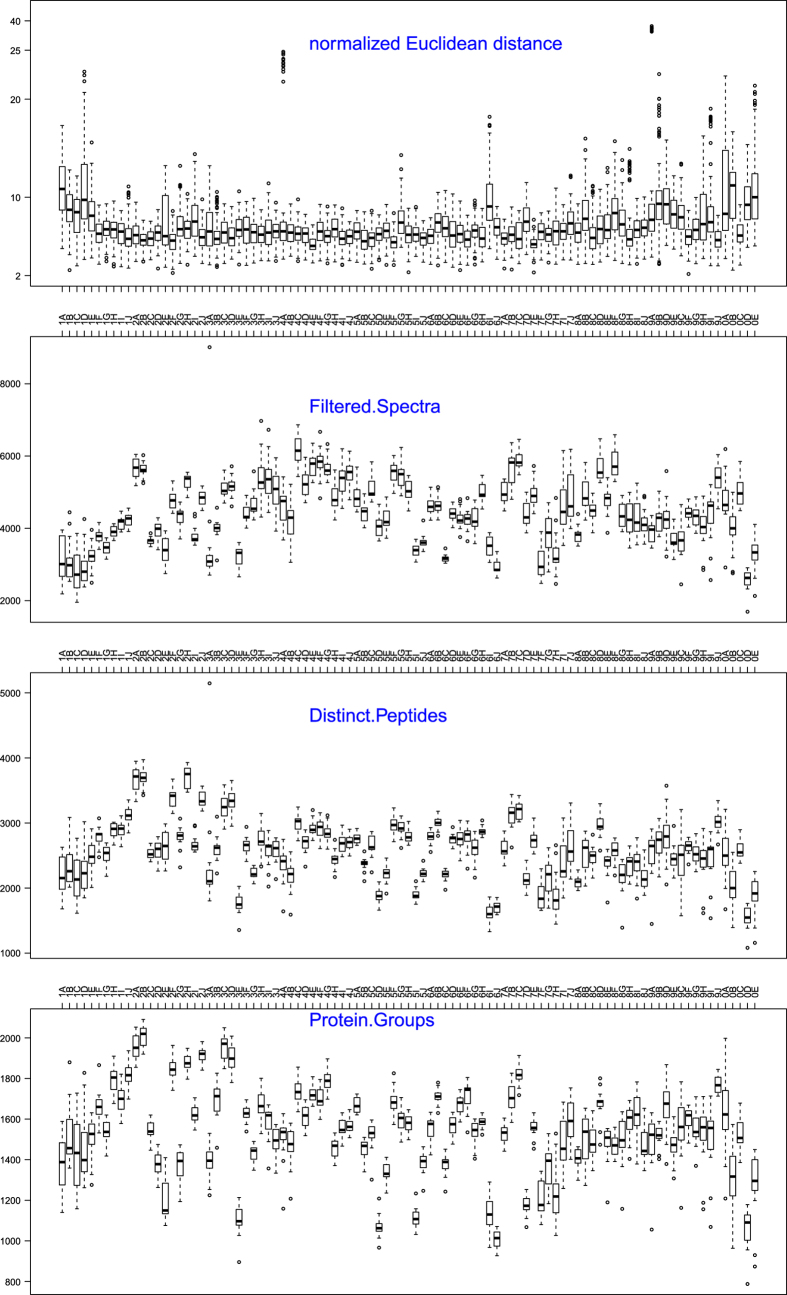
Comparison of all TCGA samples with respect to normalized Euclidean distance based on performance metrics and numbers of filtered spectra, distinct peptides and protein groups.

**Figure 6:**
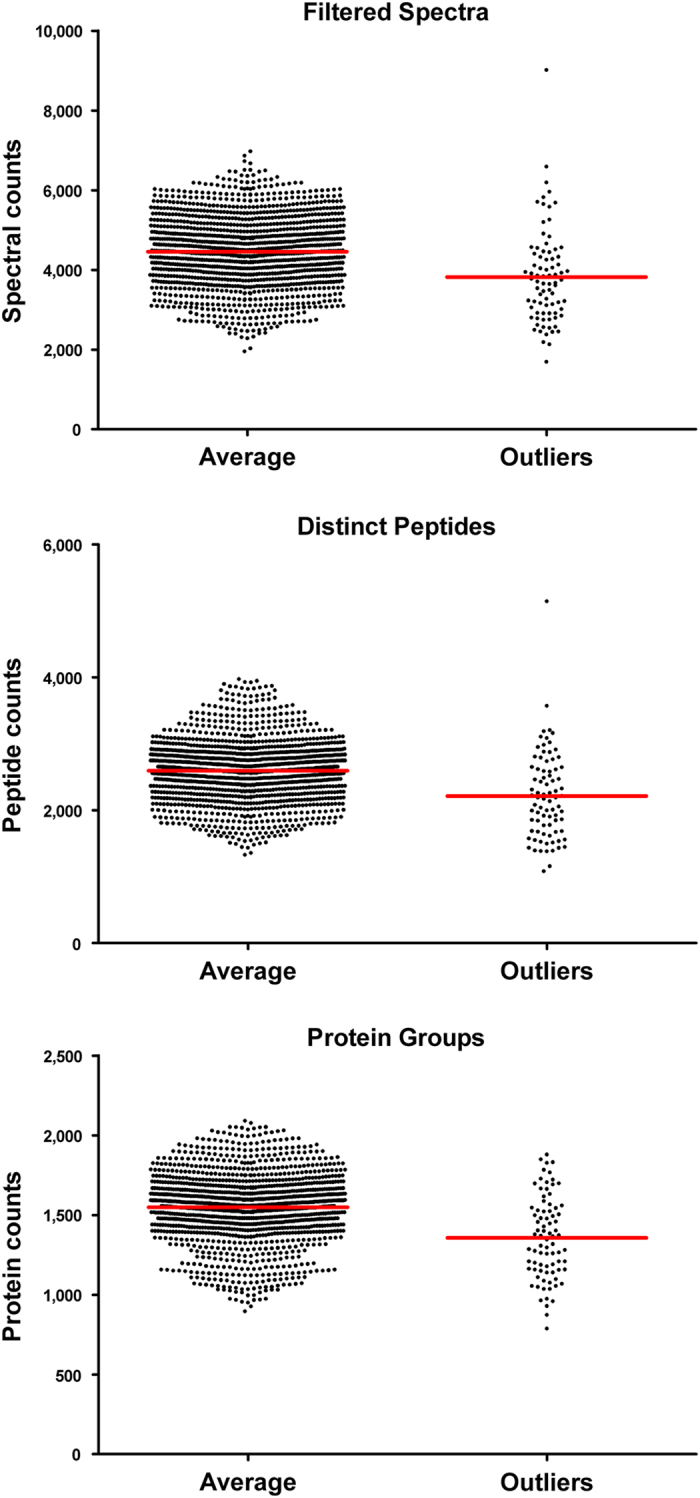
Comparison of protein identification values between LC-MS/MS experiments classified as ‘outlier’ and ‘non-outlier’ based on performance metrics. The mean values for spectral counts, peptide and protein identification were lower in the analyses classified as outliers.

Within each set of fifteen LC-MS/MS experiments, statistical scripts were used to determine the distance separating all possible pairs of the 15 fractions (Fig. 5, panel a). For most samples, the median value was close to the mean and relatively few distances were substantially lower or higher than the median. For each tumor sample, we compared Euclidian distances of each of the 15 fractions to the sample median and used the T^2^ statistic in a Chi^2^-test to identify outlier values. When we set the cut-off at *P*<0.01, 82 of the 1425 fractions are identified as outliers (indicated in blue). Figure 5 also shows the number of identified spectra, unique peptides and protein groups for each of the fractions. These plots show the variability within each sample and the variability between fractions. A 2-way ANOVA analysis of these numbers showed that more than 80% of the variability was due to differences between samples, with just 3–5% of the variability due to fraction and the remainder was the error component. Thus, differences in protein composition of the various tumor samples make up the vast majority of the difference observed in spectral counts, distinct peptides and protein groups. When we compared the search results from the 82 fractions identified as outliers by the performance metrics, these fractions had lower identifications of spectra, peptides and proteins compared to the non-outlier fractions (Fig. 6). However, individual fractions with normal or above-normal identifications were also part of this group. Thus, our conclusion from these analyses is that outliers identified with the performance metrics do not necessarily identify sub-standard fractions in this dataset, but that they may indicate sources of variability that could be better controlled in future studies.

## Usage Notes

To date, our compilation of TCGA colorectal carcinomas yielded the first large proteomic dataset for a human cancer. The use of alternative analysis strategies on this dataset have the potential to identify a larger number of proteins and protein forms. The datasets included with the original publication consisted of all instrument (RAW) files and the final end product files in the form of IDP3 database (idpDB) files. The pipeline that leads from the source data to the final IDP3 file, described in detail under ‘Methods’, represents one of the possible strategies of dealing with large proteomic datasets. In this publication, we make available all intermediate files that were generated in the process of analyzing this dataset. These include the standard and individual customized FASTA databases used for protein search, the standard spectral library, and the search result files (pepXML/mzID) from Myrimatch, Pepitome and MS-GF+ searches. These files can serve as direct input to the various other proteomic analysis tools such as the Trans Proteomic Pipeline (TPP)^33^, or commercial packages such as Scaffold.

To facilitate access to the TCGA colorectal dataset we created a Colorectal Cancer Portal in NetGestalt^34^ that encompasses data from all analyses types performed on this set of clinical specimens (http://www.netgestalt.org), including genomic, epigenomic, transcriptomic, proteomic, and clinical data. The portal also includes microarray gene expression data from the Gene Expression Omnibus GEO for five CRC cohorts; mRNA expression, copy number, mutation, and drug response data for CRC cell lines from the Cancer Cell Line Encyclopedia project; and shRNA-based high-throughput screen data for CRC cell lines from the Achilles project. The portal provides for easy data access and visualization, data integration of proteomic data with other colorectal carcinoma data types, and biological interrogation against multiple biological knowledge bases (protein interaction networks, MsigDB, KEGG etc.). Examples of such analyses include the integration of all data types for a single gene or a set of genes that allow comparisons of epigenetic, copy number, mutational, mRNA expression, and protein expression data; the query of protein networks that are associated with specific sub-types of colorectal carcinoma; and the prioritization of candidate drivers by integrating multiple types of data.

## Additional Information

**How to cite this article:** Slebos, R. J. C. *et al.* Proteomic analysis of colon and rectal carcinoma using standard and customized databases. *Sci. Data* 2:150022 doi: 10.1038/sdata.2015.22 (2015).

## Supplementary Material



Supplementary Tables
